# Hyperspectral imaging in oral oncology: a scoping review

**DOI:** 10.3389/froh.2026.1857460

**Published:** 2026-07-07

**Authors:** Shamal Kankawale, Amol Jamkhande, Gauri Oka

**Affiliations:** 1 Central Research and Publication Unit, Bharati Hospital and Research Centre, Pune, India; 2 Community Dentistry, Bharati Vidyapeeth (Deemed to be University) Dental College, Pune, India; 3 Central Research and Publication Unit, Bharati Vidyapeeth (Deemed to be University) Medical College, Pune, India

**Keywords:** deep learning, diagnostic accuracy, hyperspectral imaging, intraoperative imaging, machine learning, oral cancer, oral squamous cell carcinoma

## Abstract

**Introduction:**

Oral cancer accounts for 177,000 deaths annually. Despite advances in surgical techniques and adjuvant therapies, the five-year survival rate for oral malignancies has shown limited improvement. Hyperspectral imaging (HSI) is a new non-invasive optical modality that extends medical imaging beyond the visible spectrum. This scoping review aims to map the extent, nature, and methodological characteristics of the existing literature on the application of HSI for the detection and characterization of oral cancer. It explores the clinical applications of HSI in oral oncology, outlines the technical and analytical approaches used, and evaluates its diagnostic performance and translational readiness in clinical practice.

**Methods:**

This scoping review used the Preferred Reporting Items for Systematic Reviews and Meta-Analyses extension for Scoping Reviews (PRISMA-ScR) guidelines and followed the Population-Concept-Context(PCC) framework. A comprehensive search strategy was formulated to search across PubMed, ScienceDirect, Nature, LILACS, and Cochrane Library, and citation searching. Search was conducted by two independent reviewers using predefined inclusion and exclusion criteria. Microsoft Excel was used for data extraction. Descriptive synthesis was used to map the extent and nature of existing evidence, and narrative synthesis was used to identify patterns and methodological characteristics.

**Results:**

Out of 1,513 records identified, 15 studies were included. Most studies were published after 2017 and conducted in high-income countries. 13 studies were conducted*ex vivo*, and 2 were *in vivo*. Most studies primarily focused on oral squamous cell carcinoma. All studies used reflectance based HSI with macroscopic and microscopic systems across visible-near-infrared ranges. Machine and deep learning methods were used. Clinical applications of HSI included diagnosis, margin assessment, and segmentation. Studies showed strong diagnostic performance with high sensitivity and specificity and area under the curve values.

**Conclusion:**

HSI is a new emerging technology with applications in oral oncology. It provides a non-contact, objective and rapid alternative for margin assessment. With improved standardization, robust analytical models and clinical validation, it has the potential to be integrated into routine oral cancer management.

**Systematic Review Registration:**

https://doi.org/10.17605/OSF.IO/JQEGU.

## Introduction

Oral cancer is a significant global health issue affecting populations with lifestyle risk factors and inadequate healthcare access (1). Globally, it leads to approximately 377,000 new cases each year and about 177,000 deaths annually (2). Oral squamous cell carcinoma (OSCC) accounts for 90% of all cases, and salivary gland neoplasms account for about 5% (1). Despite advances in surgical techniques and adjuvant therapies, the five-year survival rate for oral malignancies has remained the same (3, 4). To improve patient outcomes, it is crucial to understand molecular features, size, and depth at the time of initial diagnosis (5, 6).

The current gold standard for oral cancer diagnosis relies on visual inspection and tactile palpation, followed by invasive incisional biopsy and histopathological assessment (7, 8). The process is time-consuming, invasive, and prone to inter-observer variability (6, 9). Intraoperative methods, such as frozen section (FS), are labor-intensive and may fail to reflect the full extent of the disease due to sampling errors (6).

Hyperspectral imaging (HSI), an emerging non-invasive optical modality, extends medical imaging beyond the visible spectrum (10). HSI was developed for remote sensing and Earth observations using digital imaging and spectroscopy to obtain a three-dimensional hypercube (1, 10, 11). Every pixel within this hypercube contains a continuous optical spectrum that serves as an optical fingerprint, reflecting the molecular composition and pathological state of the underlying tissue (11).

Literature highlights the potential of HSI for intraoperative margin assessment. Intraoperative margin assessment is the evaluation of surgical tissue margins during cancer resection to determine whether residual tumor cells remain at the margins. Most importantly in tongue cancer where positive or close margins occur in about 30% of the cases (12). HSI bypasses traditional autofluorescence diagnosis by providing real-time, label-free guidance to differentiate tumors from healthy tissue with high sensitivity (7). Microscopic Hyperspectral Imaging (MHI) provides a quantitative framework for pathological staging by using Euclidean distances to differentiate various OSCC stages (3).

HSI is advancing beyond traditional pixel-wise classifiers such as Support Vector Machines (SVM), Linear Discriminant Analysis (LDA), and Random Forests, which require manual engineering and Principal Component Analysis (PCA) (13). Recent research uses deep learning (DL) architectures, such as Convolutional Neural Networks (CNNs), and semantic segmentation models, such as the U-Net (11). This new technology learns detailed spatial and spectral patterns directly from the data cube, enabling it to better reduce saliva glare and handle larger data sizes.

**Figure 1:**
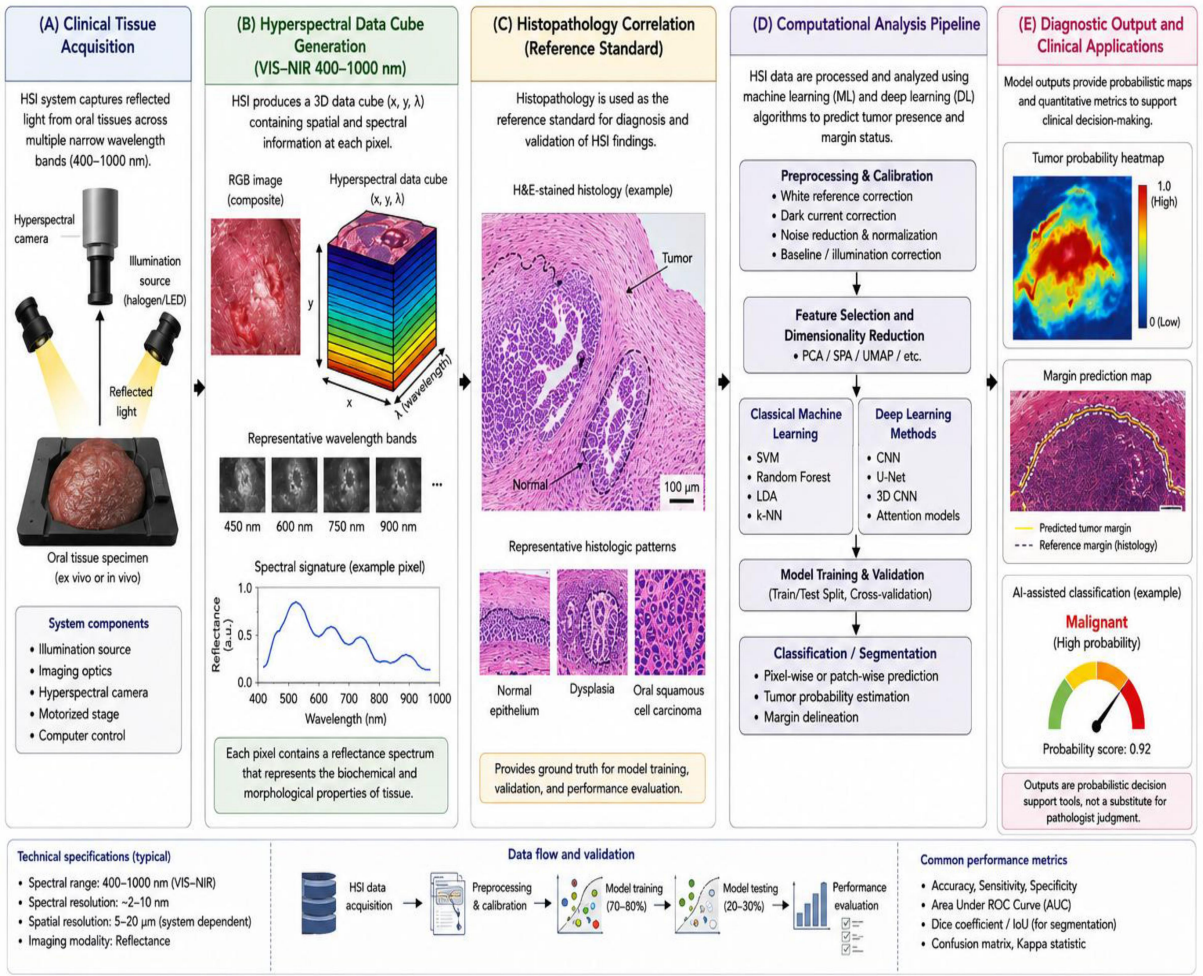
Conceptual overview of HSI workflow in oral oncology applications. The schematic illustrates the general workflow of hyperspectral imaging in oral oncology, including clinical tissue acquisition, hyperspectral data cube generation, histopathological correlation, computational analysis using machine learning (ML) and deep learning (DL) approaches, and diagnostic applications such as lesion detection, tissue characterization, and tumor margin assessment. Histopathology serves as the reference standard for validation of hyperspectral imaging findings. The conceptual framework of this figure was synthesized from previously published literature on hyperspectral imaging in oral oncology and histopathological image analysis, based on concepts synthesized by Liu et al. (14), Brouwer de Koning et al. (12), Halicek et al. (15), Ma et al. (13), Pertzborn et al. (16), Lu et al. (17), and Trajanovski et al. (18). This figure was created using Canva and ChatGPT (OpenAI) as an original conceptual schematic synthesized from the reviewed literature and does not reproduce copyrighted figures or images from previously published studies.

Given the rising advancements in the field, there is a need to map existing evidence to identify trends, evaluate methodological quality, and identify research gaps in the development and application of HSI. While previous reviews have analyzed HSI in medicine and various cancer detection, none have specifically gauged its applications within oral oncology, particularly for oral squamous cell carcinoma, surgical margin assessment and lesion characterization. This scoping review aims to systematically map the extent, nature, and methodological characteristics of the existing literature on the application of HSI for the detection and characterization of oral cancer. It seeks to 1) summarize the clinical applications of HSI in oral oncology, (Figure 1) 2) outline the technical and analytical approaches used, and 3) evaluate the diagnostic performance and translational readiness of HSI in clinical practice.

## Methodology

This scoping review used the Preferred Reporting Items for Systematic Reviews and Meta-Analyses extension for Scoping Reviews (PRISMA-ScR) guidelines (19). The study followed the Population-Concept-Context (PCC) framework (20). Here, the **p**opulation refers to human oral mucosal tissues with premalignant and malignant epithelial lesions. The **c**oncept is the use of hyperspectral imaging to detect and characterize oral mucosal lesions. The **c**ontext is clinical and translational oral health research. This review protocol was registered on Open Science Framework (OSF) (21).

### Study selection

Studies were eligible if they studied the human oral mucosal tissues with premalignant and/or malignant epithelial lesions. Assessment of the lesions was done using HSI, with or without computational analysis, for detection, diagnosis, characterization, margin assessment, or monitoring. Histopathology or a clinically accepted diagnostic method served as the reference standard.

Studies involving head and neck malignancies were included when oral cavity tissues, oral mucosal lesions or clinically relevant oral oncology applications were analyzed.

Studies were excluded if they focused exclusively on non-oral head and neck sites, investigated non-neoplastic conditions or employed non-HSI imaging tools. Studies that were purely methodological, engineering-focused, or related to device development without a clinical diagnostic application were also not considered. Reviews, editorials, conference abstracts lacking full data, and studies involving non-human subjects were also excluded Table 1.

**Table 1:** Inclusion and exclusion criteria used for screening of the articles.

Inclusion criteria	Exclusion criteria
Human oral mucosal lesions	Non-oral head and neck lesions
Premalignant/malignant lesions	Non-neoplastic conditions
HSI-based assessment	Non-HSI modalities
Diagnostic/ margin/ monitoring applications	Engineering-only studies
Histopathology or clinically accepted reference standard	Animal studies, reviews, editorial, abstracts

### Search strategy

A search was conducted across five databases: PubMed, ScienceDirect, Nature, LILACS, and Cochrane Library. Backward and forward citation searching (snowballing) was also performed. The search strategy combined MeSH terms and free-text keywords related to HSI and oral or dental tissues. There were no restrictions on the date of publication. No restrictions were applied on language and studies in languages other than English were translated using browser-based translation tools. The complete database-specific search strings, search dates and retrieval counts are outlined in Supplementary 1.

**Figure 2:**
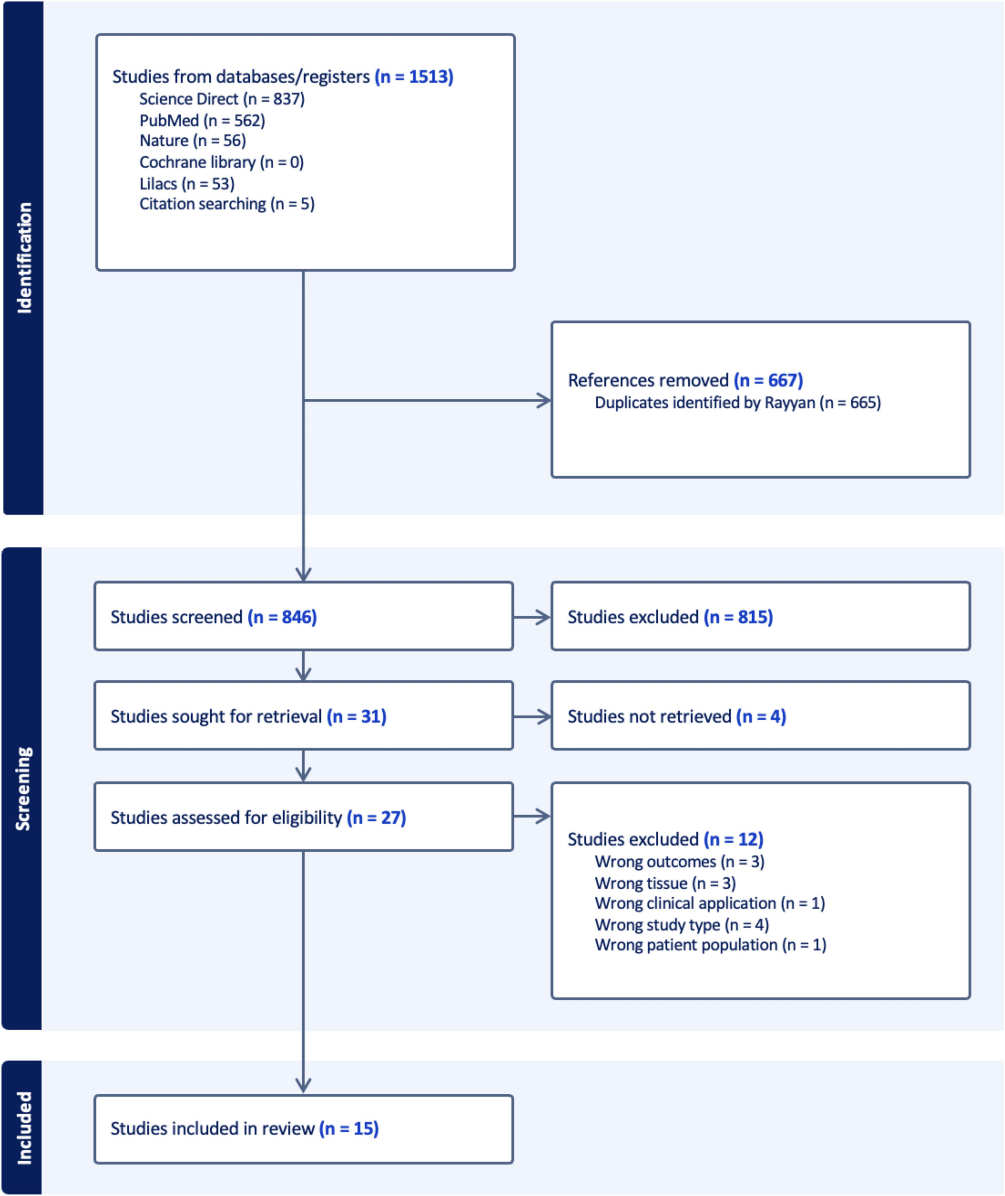
PRISMA-ScR flow diagram. Flow diagram showing the study selection process. 1,513 records were identified and 15 studies were included.

All studies from the databases were imported to Rayyan for screening and deduplication. SK conducted title and abstract screening using the predefined inclusion criteria. SK, GO, and AJ conducted independent full-text screening, and any conflicts were resolved through discussion. Reasons for exclusion at full-text screening are presented in the PRISMA flow diagram (Figure 2).

### Data extraction

Data from the included studies were extracted by SK and AJ independently using Microsoft Excel (version 16) under the headings of study characteristics (author, year, country); study design and imaging setting (*in vivo*/*ex vivo*; microscopic/ macroscopic); clinical application of HSI; anatomical site and type of lesion; HSI modality and analytical approach, reference standard and validation strategy and key reported outcomes. Any conflicts were resolved through discussion.

### Data synthesis

Descriptive synthesis was used to map the extent and nature of existing evidence. Studies were grouped primarily according to the clinical application of HSI. Narrative synthesis was used to identify patterns and key methodological characteristics, including imaging modality, acquisition setting, analytical approach, and validation strategy. Given the heterogeneity of the studies, quantitative analysis was not undertaken.

## Results

### Study selection

A comprehensive search was conducted across five databases: PubMed, ScienceDirect, Nature, LILACS, and Cochrane Library and citation searching identified 1,513 articles. After deduplication in Rayyan, 846 articles were screened based on title and abstract. Full-text screening was then done for 31 studies, and 15 were included in this scoping review. The selection process for the studies, along with the reasons for exclusion are outlined in the PRISMA-ScR flow diagram (Figure 2).

### Study characteristics

Table 2. The included studies were conducted across seven countries. The majority was seen in high-income countries from North America and Europe. Most studies took place in the United States (*n* = 4) (1, 8, 22, 23), followed by Germany (*n* = 3) (16, 24, 25), China (*n* = 2) (3, 14), the Netherlands (*n* = 2) (12, 18), Israel (*n* = 2) (9, 26), Taiwan (*n* = 1) (27), and Austria (*n* = 1) (4).

**Table 2:** Study characteristics of the included studies.

Study (author, year)	Country	Study design	Clinical application	Sample size	Lesion type	Anatomical site	HSI modality	Spectral range (nm)	Imaging setup	Analysis method	Reference standard	Validation	Key outcomes	Clinical readiness
Liu et al., 2011 (14)	China	*In vivo* feasibility	Diagnosis	65 tumors	OSCC	Tongue	Reflectance HSI	600–1,000	Macroscopic (*in vivo*)	Sparse representation (ML)	Histopathology	Internal split	Accuracy 96.5%	Exploratory
Brouwer de Koning et al., 2019 (12)	Netherlands	*Ex vivo* feasibility	Margin assessment	14 patients	OSCC	Tongue	Diffuse reflectance HSI	400–1,700	Macroscopic (*ex vivo*)	Neural network (DL)	Histopathology	Cross-validation	AUC 0.93	Pilot
Trajanovski et al., 2020 (18)	Netherlands	*Ex vivo* feasibility	Tumor detection/segmentation	14 patients	OSCC	Tongue	Reflectance HSI	400–1,700	Macroscopic (*ex vivo*)	U-Net–based segmentation (DL)	Histopathology	Leave-one-patient-out CV	Dice ≈0.89; AUC ≈0.92	Pilot/near-clinical
Ou-Yang et al., 2015 (27)	Taiwan	*Ex vivo* feasibility	Diagnosis (biopsy-based)	68 biopsies (34 patients)	OSCC	Oral cavity	Reflectance + fluorescence HSI	400–1,000	Microscopic (biopsy slides)	Morphological + spectral features (ML)	Histopathology (H&E)	Internal split	Sensitivity 90 ± 4.53%; specificity 87.8 ± 5.21%	Exploratory
Mittermair et al., 2025 (4)	Austria	Retrospective feasibility	Biomarker characterization	50 patients	OSCC	Oropharynx	VIS–NIR reflectance HSI	500–1,000	Microscopic (FFPE sections)	PCA-based spectral analysis	Histopathology + IHC	Internal comparison	Correlation with IHC (*ρ* ≈0.67)	Exploratory
Sudri et al., 2025 (9)	Israel	*Ex vivo* feasibility	Diagnosis/characterization	49 samples	Salivary gland neoplasms	Salivary glands	Contrast-enhanced reflectance HSI	400–900	Microscopic (FFPE sections)	Spectral intensity analysis	Histopathology + IHC	Internal validation	Sensitivity 79%; specificity up to 100%	Exploratory
Boehm et al., 2025 (24)	Germany	*Ex vivo* feasibility	Margin assessment	32 patients	HNSCC	Oral cavity & pharynx	Reflectance HSI	470–780	Macroscopic (*ex vivo*)	CNN and GNN-based DL	Histopathology	Leave-one-patient-out CV	Accuracy up to 89%; AUC up to 0.95	Pilot/near-clinical
Felicio-Briegel et al., 2024 (25)	Germany	*In vivo* feasibility	Postoperative perfusion monitoring	14 patients	HNSCC	Oral cavity	Reflectance HSI	500–1,000	Macroscopic (*in vivo*intraoral)	Quantitative perfusion indices (StO₂, THI, NPI, TWI)	Clinical exam + Doppler	Internal longitudinal comparison	Stable perfusion parameters in viable flaps	Pilot/near-clinical
Ma et al., 2023 (13)	USA	*Ex vivo* feasibility	Diagnosis (histopathology support)	20 patients (26 slides)	OSCC	Head & neck	Microscopic HSI	470–720	Microscopic (H&E slides)	PCA-based nuclei segmentation + SVM & CNN (DL)	Histopathology (pathologist annotations)	Leave-one-patient-out CV	AUC up to 0.93; accuracy up to 0.89	Exploratory
Pertzborn et al., 2023 (16)	Germany	*Ex vivo* feasibility	Margin assessment	7 patients (23 tissue sections)	OSCC	Oropharynx/oral cavity	Reflectance HSI	500–1,000	Microscopic (unstained frozen sections)	3D CNN and ResNet-based classifiers (DL)	Histopathology (H&E, pathologist annotation)	Cross-validation	Accuracy 0.76; specificity 0.89; sensitivity 0.48	Pilot/near-clinical
Halicek et al., 2018 (15)	USA	*Ex vivo* feasibility	Diagnosis/optical biopsy	21 patients (60 tissue specimens)	OSCC	Oral cavity	Reflectance HSI	450–900	Macroscopic (fresh surgical specimens)	CNN-based deep learning (patch-based classification)	Histopathology (H&E)	Leave-one-patient-out CV	AUC 0.82; accuracy 81%; sensitivity 81%; specificity 80% (SCC vs. normal)	Pilot/near-clinical
Lu et al., 2019 (17)	USA	*Ex vivo* feasibility	Margin assessment	6 patients (60 images)	HNSCC	Oral cavity	Label-free reflectance HSI	450–900	Macroscopic (fresh *ex vivo*specimens)	Spectral feature extraction + ML classifiers	Histopathology (H&E-annotated margins)	Leave-one-patient-out CV	Accuracy ≈90%; sensitivity ≈89%; specificity ≈91%	Pilot/near-clinical
Fei et al., 2017 (8)	USA	*Ex vivo* pilot feasibility	Tumor margin assessment	16 patients	HNSCC	Oral cavity	Label-free reflectance HSI	450–900	Macroscopic (*ex vivo*surgical specimens)	Ensemble linear discriminant analysis (ML)	Histopathology (H&E)	Internal (within-patient training/testing)	Accuracy 90%; sensitivity 89%; specificity 91%; AUC ≈0.94 (oral cavity)	Pilot
Hirshberg et al., 2017 (26)	Israel	*Ex vivo* feasibility	Diagnosis/lesion discrimination	30 cases (5 controls, 15 dysplasia, 10 OSCC)	Dysplasia & OSCC	Oral cavity	Contrast-enhanced HSI (gold nanorods)	∼400–900 (analysis at 660)	Microscopic (FFPE tissue sections)	Spectral intensity analysis; ROC analysis	Histopathology (H&E)	Internal validation	AUC up to 0.97 for benign/mild vs. advanced lesions	Exploratory
Zhang et al., 2025 (3)	China	*Ex vivo* feasibility	Pathological staging/diagnosis	80 sections	OSCC	Tongue	Microscopic reflectance HSI	370–1,050	Microscopic (H&E slides)	ELM, SVM, PLS-DA with dimensionality reduction via PCA and SPA	Histopathology (H&E)	Internal validation	SPA-ELM: Accuracy 100%, Sensitivity 100%, Specificity 100%, Kappa 1.0000, AUC 0.9949	Exploratory/Proof-of-concept

Table showing study characteristics of the included studies.

OSCC, oral squamous cell carcinoma; HNSCC, head and neck squamous cell carcinoma; SVM, support vector machine; PLS-DA, partial least squares discriminant analysis; ELM, extreme learning machine; ML, machine learning; DL, deep learning; AUC, area under the curve; ROC, receiver operating characteristic; SPA, successive projections algorithm, H&E, hematoxylin and eosin; CNN, convolutional neural network; CV, cross validation; GNN, graph neural network; IHC, immunohistochemistry; FFPE, formalin fixed paraffin embedded; StO_2_, tissue oxygen saturation; THI, tissue hemoglobin index; NPI, near infrared perfusion index; TWI, tissue water index; HSI, hyperspectral imaging. Exploratory studies were defined as proof-of-concept studies assessing the technical feasibility of HSI. Pilot studies were defined as preliminary clinical investigations evaluating diagnostic performance in small patient cohorts. Near-clinical studies were defined as studies conducted in clinically relevant settings with workflows approximating intended clinical use, but without routine implementation or large–scale validation.

The studies were published between 2012 and 2025. The earliest identified study was conducted by Liu et al. in 2012 in China (14). Publications increased after 2017, with a peak occurring in 2025 (*n* = 4) (3, 4, 9, 24).

All included studies used feasibility study designs. Most studies (*n* = 13; 86.7%) (1, 3, 4, 8, 9, 12, 16, 18, 22–24, 26, 27) were*ex vivo*, analyzing excised specimens, histological tissue sections, or biopsy samples. Only two studies (14, 25) incorporated *in vivo*imaging in humans. Liu et al. (14) examined 65 oral tumors, and Felicio-Briegel et al. (25) studied 14 patients with reconstructed head and neck defects.

Sample sizes in the included studies ranged from 7 to 50 participants in patient-based cohorts and from 14 to 80 in specimen-based or section-based approaches. All studies were based in a single institute. All studies were in pilot or exploratory stages.

### Lesion types and clinical applications

OSCC was the predominant lesion (*n* = 9) (1, 3, 4, 12, 14, 16, 18, 22, 27). One study examined epithelial dysplasia alongside OSCC, whereas most focused on malignant lesions rather than premalignant or benign oral mucosal pathology (26). Head and neck squamous cell carcinoma was identified in four studies, which included oral cavity tissues, oral mucosal lesions or clinically relevant oral oncology applications relevant to the review inclusion criteria (8, 23–25).

Clinical applications were categorized as:

1. Diagnostic (*n* = 7) (3, 9, 13, 14, 22, 26, 27): to differentiate malignant lesions from normal tissue, dysplastic lesions, or benign pathology
2. Surgical margin assessment (*n* = 5) (8, 12, 16, 23, 24): to identify tumor-infiltrated regions to guide intraoperative decision making.
3. Tumor detection and segmentation (*n* = 1) (18): one study used deep learning-based semantic segmentation to delineate tumor boundaries within hyperspectral images.
4. Other applications (*n* = 2) (4, 25): these include biomarker characterization and postoperative perfusion monitoring

### HSI modalities and technical specifications

All studies used reflectance based HSI. One used both reflectance and fluorescence HSI (27), and two used contrast-enhanced HSI using gold nanorod conjugates to improve lesion discrimination (9, 26). Label-free reflectance systems were used for fresh surgical specimens (8, 23). Contrast-enhanced protocols using gold nanorods were applied to *ex vivo*sections to enhance optical contrast in dysplasia and salivary gland neoplasms (9, 26).

The imaging setup included spatial resolution and clinical workflow considerations. They were categorized into:

1. Macroscopic HSI systems (*n* = 8) (8, 12, 14, 18, 22–25): they were designed to capture images of fresh surgical samples, *in vivo*tissues, or large tissue sections at millimeter-scale resolution.
2. Microscopic HSI systems (*n* = 7) (3, 4, 9, 13, 16, 26, 27): were used, along with standard lab methods, to collect color data from slides, with or without stains.

Across the included studies, HSI operated in the visible to near-infrared range with lower limits between 370 and 600 nm and upper limits between 780 and 1700. Tongue and margin studies using diffuse reflectance covered 400 to 1700nm (12, 18), and microscopic systems on histological slides mostly used narrower bands within 400 to 1,000 nm or 470 to 720 nm (13, 27).

Macroscopic systems captured *in vivo*wide-field images of tongues, free flaps, or fresh surgical margins at millimeter-scale resolution. Microscopic systems were used in pathology workflows to FS, FFPE tissue, or hematoxylin and eosin (H&E) stained slides at cellular resolution.

### Analytical methods and validation

Analytical methods ranged from traditional feature-based machine learning (ML) to complex deep learning models. Preprocessing included noise reduction (median filtering or Savitzky-Golay smoothing), normalization using white or dark references, and dimensionality reduction with principal component analysis (PCA) or successive projections algorithm (SPA).

Validation used cross-validation with 60/20/20 train/validation/test splits, leave-one-out inter-patient testing, and ROC analysis reporting area under the curve (AUC), accuracy, sensitivity, specificity, and Kappa statistics. Ground truth derived from registered H&E-stained histopathology, with pathologist-annotated tumor margins aligned to HSI.

### Diagnostic and margin-assessment performance

For tumor vs. non-tumor discrimination on biopsy sections or stained slides, sensitivity and specificity were mostly in the 80%–90% range, with AUC values above 0.9.

ML and DL analysis methods demonstrated strong diagnostic performance. Zang et al. (3) achieved 100% accuracy, sensitivity, and specificity for staging classification using ML, while Trajanovski et al. reported an AUC of 0.92 for tumor detection using DL (18). Five ML DL studies evaluated surgical margin assessment. CNN-based classification (28) used by Halicek et al. on 60 oral cancer tissue specimens achieved an accuracy of 81% (22). Similarly, Pertzborn et al. reported an accuracy of 76% across 23 tissue sections (16).

Studies conducted using Gold Nanoparticles by Boehm et al. and Hirshberg et al. revealed clear differentiation between benign, dysplastic, and malignant tissue (9, 26). The two *in vivo*studies reported a 96.5% tumor detection rate, demonstrating the feasibility of postoperative perfusion monitoring (14, 25).

### Cross-cutting patterns

Methodological and application trends were observed across all included studies. All used reflectance-based hyperspectral imaging systems operating in the visible-to-near-infrared spectrum. Machine learning and deep learning were widely used in studies focusing on diagnostic discrimination and surgical margin assessment (1, 3, 8, 12, 14, 16, 18, 23, 24, 27).

## Discussion

### Summary of main findings

This review of 15 pilot and exploratory studies, analyzed the applications of HSI for detecting and characterizing oral mucosal lesions. The analysis inferred that the field is undergoing a critical transitional phase. The number of publications has increased since 2017, with four publications in 2025 (3, 4, 9, 24). This growing interest in HSI applications within oral health may be due to recent advancements in computational imaging, machine learning, and intraoperative guidance technologies.

The majority of the evidence (86.7%) is derived from *ex vivo*studies that used biopsy samples, excised specimens, or histological tissues. The eligibility criteria included premalignant lesions and benign oral mucosal pathology and the review found OSCC and tongue cancer were the primary clinical focus. HSI shows strong diagnostic performance in all studies, with sensitivity and specificity ranging from 80% to 90%, and AUC values exceeding 0.90 in controlled microscopic settings. The highest diagnostic performance was seen in a microscopic HSI study, which benefits from controlled imaging conditions and a direct correlation with histopathologic findings (3).

Even though the results are promising, most studies were conducted with small sample sizes in single-center settings. Macroscopic HSI was developed for intraoperative applications and exhibited greater variability in sensitivity for margin detection, mainly in heterogeneous or thin resection edges. This variability is attributable to the dynamic nature of the oral environment. The existing literature reports the use of reflectance HSI in the visible-to-near-infrared spectrum (400–1700nm). These findings underscore HSI's capacity to detect malignancies that are not visible to the naked eye by integrating spatial and spectral data.

Though a risk of bias assessment was not performed for this review, the included studies showed several recurring methodological limitations. Most studies were pilot feasibility or exploratory with a small sample size, recruited from a single centre, with no external validation and relied on internal cross validation techniques. Heterogeneity in spectral acquisition parameters, preprocessing methods, and analytical pipelines reduced comparability across studies.

### Comparison with existing literature

The diagnostic accuracy of HSI compares with current clinical techniques such as visual inspection and palpation used for differentiating malignant from benign tissues (8, 29). Existing methods are subjective and operator-dependent often resulting in close surgical margins in 10%–30% of OSCC cases (30). Intraoperative FS analysis helps to reduce this risk (30). FS accuracy ranges from 71% to 92%, with sensitivity for detecting close margins as low as 34% to 77% due to sampling errors and tissue distortion during processing (28, 30). The process requires 20–60 min which prolongs the time of surgery (4).

HSI may provide more detailed and objective information than standard red, green, blue (RGB) imaging (15). RGB cameras capture only broad colour bands, whereas HSI cameras capture hundreds of narrow spectral bands (17). This capability enables the detection of subtle biochemical changes and features of malignancy, such as variations in haemoglobin levels and oxygenation, which are not visible with conventional imaging (17, 31).

Other optical diagnostic tools like autofluorescence imaging that detect endogenous fluorophore changes associated with dysplasia and malignancy, are compromised by inflammation and tissue diversity showing variable sensitivity and specificity (32). Optical coherence tomography (OCT) is limited by shallow penetration depth and restricted field of view despite its ability to provide high resolution cross sectional imaging of tissue microarchitecture (33). In addition, narrow band imaging (NBI) enhances visualization of mucosal vascular patterns and superficial epithelial alterations but relies on operator interpretation and expertise (10). HSI simultaneously captures both spatial and spectral information across a broad wavelength range, enabling detailed tissue characterization. It also enables non-contact, wide-field imaging, capturing biochemical and morphological tissue characteristics through spectral signatures.

Label-free HSI may offer an advantage over fluorescence-based methods that utilize dyes such as 2-NBDG and proflavine (23). HSI relies on natural tissue signals to generate a consistent optical profile. Fluorescence signals can be influenced by factors such as keratin, which reduces reliability in certain OSCC cases (6, 30).

Unlike point-probe spectroscopy, which can examine only one point at a time HSI can scan the entire surgical surface reducing the possibility of sampling errors (29, 34). It enables real-time assessment of large tissues and improves the detection of residual tumor (33). The rapid and non-contact properties of HSI support its role as an optical biopsy tool. It can complement the existing intraoperative methods by providing a more comprehensive evaluation of surgical margins.

### Technical and biological mechanisms

HSI captures subtle changes in tissue chemistry that happen when cells become cancerous. Researchers have identified a characteristic of hemoglobin's light absorption in the 450–600 nm range, with a peak around 560–565 nm (17). This peak is associated with oxygenated hemoglobin, which is linked to OSCC-related vascular changes. Cancerous tissue typically shows increased metabolism and enhanced blood vessel formation. This reinforces the association between light-absorption patterns and the presence of OSCC (8, 35). Normal tissues exhibit higher near-infrared (NIR) absorption, indicating greater fat, collagen, and water content than cancerous tissues (8, 35).

NIR (950–1700nm) is more suited for surface-level tissues where blood absorption is negligible (12). The visible light range of 650–900 nm offers greater optical depth, making it more effective for identifying sub-surface tumor pockets up to 3–5 mm deep. This is essential for evaluating close surgical margins (12).

Studies are undergoing a major shift from ML methods, such as SVMs and LDA, to DL methods, such as CNNs. These are better suited for medical HSI, as they can integrate spectral signatures with spatial morphological features, such as nuclear shape and chromatin condensation, which are vital for histopathological diagnosis (1, 28, 36).

ML approaches such as SVM, LDA, PCA and random forest classifiers generally rely on handcrafted spectral feature extraction and dimensionality reduction techniques prior to classification (37). This approach shows strong performance in smaller and controlled datasets with studies reporting more than 80% accuracies. DL approaches like CNNs, U-Net architectures, ResNet-based models and semantic segmentation frameworks enabled automated feature extraction and improved integration of both spatial and spectral information from hyperspectral datasets (18, 32). They showed better performance for tissue segmentation, tumor boundary delineation, and large-scale image analysis with an AUC of more than 0.90. DL models require vast data and powerful computers to work efficiently (38).

### Clinical implications

HSI can act as an optical biopsy tool, offering real-time, non-invasive assessment of tissue properties without the need for excision of the tissue or ionizing radiation (10). A key benefit of HSI is its time efficiency. It completes a tissue analysis in about 2 min (30). This fast-tracked response could support intraoperative cancer probability mapping, reduce surgery time, and help surgeons achieve complete tumor clearance while preserving healthy tissue.

HSI may prove more valuable in minimally invasive laparoscopic and robotic procedures, where tactile examination is limited or not possible. It can serve as a virtual biopsy method by identifying pixels or tumor foci. This enables wide-area tissue characterization and improves the detection of infiltrated cells missed out by traditional sampling (15).

#### Limitations of included studies

##### Predominance of *ex vivo*studies

Since most existing studies have trained algorithms on fixed, excised, or stained tissues, their applicability in the dynamic environment of living tissue, with variability in lighting conditions, saliva contamination, tissue motion and operator handling remains uncertain.

##### Lack of multicenter validation

Most currently available HSI systems remain research-grade and have not yet undergone large-scale multicenter clinical validation.

##### Absence of standardized protocols

HSI systems are not yet standardized and remain largely research-grade. This reduced the generalizability of the studies.

##### Additional barriers to clinical translation

There are multiple challenges for HSI transition in clinical translation. A few practical barriers include device portability, high acquisition costs, lengthy processing pipelines, the requirement of specialized computational infrastructure, large hyperspectral data storage demands and challenges integrating HSI systems into existing surgical workflows.

##### Limitations of the review

Since the studies included in the review are highly heterogeneous, this limits quantitative analysis. The heterogeneity is likely due to variations in analytical approaches and validation strategies. Since HSI requires high infrastructure and complex systems, most research on it is conducted in high-income countries. This limits our knowledge of its functioning across different healthcare settings.

##### Future research directions

Building large, multi-institutional spectral databases is important for analyzing variability between populations. Standard protocols and interoperability across HSI systems remain a major challenge for multicentre adoption. There is a need to standardize spectral resolution, illumination sources, calibration procedures, preprocessing pipelines, and annotation strategies. This approach can help lower hardware costs, reduce data size, and speed up data collection. Large-scale *in vivo*clinical trials are needed to prove that hyperspectral imaging is reliable in the operating room. General standardization of the procedure will be essential for training artificial intelligence models and enabling external validation.

## Conclusion

HSI is a new technology introduced in oral oncology that provides an objective, non-contact, and fast alternative to traditional margin assessment. It provides real-time biomarker information during care, potentially reducing inadequate surgical margins and improving survival rates and quality of life for people with oral cancer. With better hardware standards, reliable DL systems, and a global effort to validate its use in real patients, HSI has the potential to be integrated into routine clinical workflows for oral cancer management.

## Data Availability

The original contributions presented in the study are included in the article/Supplementary Material, further inquiries can be directed to the corresponding author.
