# Supplementary material for: Hyperspectral imaging in oral oncology: a scoping review
Source: Front Oral Health. 2026 Jul 7;7:1857460. doi: 10.3389/froh.2026.1857460 (PMC13385111; doi:10.3389/froh.2026.1857460)
Supplement: Supplementary file 1 [file Table1.docx]

#### Hyperspectral Imaging For Detection And Characterization Of Oral Mucosal Lesions: A Scoping Review

| **Date** | **Database** | **Search string** | **Result** |
| --- | --- | --- | --- |
| 25/01/2026 | PubMed | ("hyperspectral imaging" OR "hyper-spectral imaging" OR hyperspectral OR "spectral imaging") AND (dentistry OR dental OR oral OR "oral health" OR gingiva OR gingival OR enamel OR dentin OR dentine OR "oral mucosa") | 252 |
|  |  | (hyperspectral imaging) AND (oral mucosa) | 8 |
|  |  | (hyperspectral imaging) AND (oral cancer) | 41 |
|  |  | ( "hyperspectral imaging" OR "hyper-spectral imaging" OR hyperspectral OR "spectral imaging" ) AND ( oral OR "oral mucosa" OR "oral lesion*" OR "oral cancer" OR "oral squamous cell carcinoma" OR OSCC OR dysplasia OR "oral potentially malignant disorder*" OR leukoplakia OR erythroplakia OR gingiva OR gingival ) | 261 |
| 25/01/2026 | ScienceDirect | hyperspectral imaging and oral cancer | 557 |
|  |  | hyperspectral imaging and oral lesions | 280 |
| 25/01/2026 | Cochrane | hyperspectral imaging and oral cancer | 0 |
|  |  | hyperspectral imaging and oral lesions | 0 |
| 25/01/2026 | Nature | hyperspectral imaging and oral cancer | 56 |
| 25/01/2026 | LILACS | hyperspectral imaging and oral cancer | 40 |
|  |  | hyperspectral imaging and oral lesions | 13 |
| 26/01/2026 | Citation searching/ Snowballing |  | 5 |
|  |  |  | 1513 |

Total uploaded - 1513, deleted duplicate - 667, Tiab - 846
